# Telemedicine during COVID-19 in India—a new policy and its challenges

**DOI:** 10.1057/s41271-021-00287-w

**Published:** 2021-05-19

**Authors:** Sambit Dash, Ramasamy Aarthy, Viswanathan Mohan

**Affiliations:** 1 Department of Biochemistry, Melaka Manipal Medical College, Manipal Academy of Higher Education, Manipal, Karnataka India; 2 Madras Diabetes Research Foundation, No 4, Conran Smith Road, Gopalapuram, Chennai, 600086 India; 3 Dr. Mohan’s Diabetes Specialities Centre, No. 6 B, Conran Smith Road, Gopalapuram, Chennai, 600086 India

**Keywords:** Telemedicine, Privacy, Data use, Internet infrastructure, Competencies, Access, Policy

## Abstract

During the COVID-19 pandemic, a countrywide lockdown of nearly twelve weeks in India reduced access to regular healthcare services. As a policy response, the Ministry of Health & Family Welfare which exercises jurisdiction over telemedicine in India, rapidly issued India’s first guidelines for use of telemedicine. The authors argue that: guidelines must be expanded to address ethical concerns about the use of privacy, patient data and its storage; limited access to the internet and weaknesses in the telecom infrastructure challenge widespread adoption of telemedicine; only by simultaneously improving both will use of telemedicine become equitable; Indian medical education curricula should include telemedicine and India should rapidly extend training to practitioner. They determine that for low- and middle-income countries (LMIC), including India, positive externalities of investing in telemedicine are ample, thus use of this option can render healthcare more accessible and equitable in future.

## Introduction

As a response to the COVID-19 pandemic, the Indian government imposed a strict countrywide lockdown. Then, out-patient departments in most government and private medical colleges either shut or curtailed availability of services [1]. Reports from countries affected more seriously by the pandemic prompted this response in India. Others had learned that closed environments, particularly hospitals, facilitated secondary transmission of the coronavirus. Hospitals plausibly functioned as sources of 'super spreading' events; to contain the spread, hospitals closed out-patient departments and other non-emergency units [2]. In India (and likely in other low- and middle-income countries) physical access to healthcare was already limited along with other dire limitations like unavailability of adequate healthcare human resource and its skewed distribution, low affordability, massive information asymmetry and poor health awareness and thus, closure of these services exacerbated a shortage of services [3].

India responded, as did many other countries worldwide, with telemedicine and other digital health technologies. Within a week of the close of hospital out-patient departments, several healthcare facilities commenced telemedicine services. Providers included large private hospitals as well as individual practitioners. Union government institutions and the State-funded ones promptly offered services by electronic means [4, 5]. Just after the closure of hospital units, the Ministry of Health & Family Welfare established the country’s telemedicine policy guidelines (25 March 2020) [6].

## History of Telemedicine in India

The World Health Organization (WHO) defines telemedicine as,

the delivery of healthcare services, where distance is a critical factor, by all healthcare professionals using information and communication technologies for the exchange of valid information for the diagnosis, treatment and prevention of disease and injuries, research and evaluation, and for the continuing education of healthcare providers, all in the interests of advancing the health of individuals and their communities [7].

The Indian Space Research Organization (ISRO) initiated telemedicine in India in 2001, starting with the Telemedicine Pilot Project. The Health Ministry established a National Telemedicine Taskforce in 2005, sharing jurisdiction with the Ministry of Health and Family Welfare (MoHFW). Additional national programs in India include the Integrated Disease Surveillance Project (IDSP), the National Cancer Network (ONCONET), the National Rural Telemedicine Network, the Digital Medical Library Network, and the National Medical College Network that links medical colleges with the primary purpose of e-learning [8].

Until emergence of the COVID-19 pandemic, India had not implemented telemedicine on a large scale, and the early attempts had not all been successful. Nor was telemedicine clearly legal before issuance of the 25 March 2020 guidelines. Several judicial orders in India impeded the practice of telemedicine. The public questioned the usefulness of telemedicine after a top court in one of the largest states of India, Maharashtra, upheld criminal negligence charges for a case that involved consultation over the telephone after which the patient lost her life [9]. Thus, a lack of clear policy or legislation and a ruling of criminal negligence left the future of telemedicine uncertain in India until COVID-19 brought it into sharp focus.

## Telemedicine Guidelines during COVID-19

By issuing guidelines for the practice of telemedicine, The Board of Governors of the Medical Council of India (MCI), the erstwhile medical education regulator in India that prepared them in consultation with the premier planning body, the NITI Aayog (National Institution for Transforming India), has attempted to fill an important gap: lack of legislation and a framework for ethical practice of telemedicine [5]. The guidelines list video, audio, and texting as three modes of communication and outline the provisions for their use of by practitioners, including limitations.

The guidelines lack clarity about privacy and data usage, for patients and practitioners. They place the onus entirely on doctors to maintain records of all exchanges of communication between themselves and patients. The guidelines do not yet specify duration for storing data nor limits to further use of that data. The guidelines simply require the practitioner to be aware of the data protection and privacy laws and follow them. Privacy concerns arise as details, including a patient’s address and other 'reasonable' identification, is required to be recorded by the practitioner. The guidelines explain the concepts of implicit and explicit consent—but a mere initiation of a telemedicine consultation by an individual is considered as implicit consent. The guidelines need to elaborate more on consent in a teleconsultation and ways to obtain and record it. The guidelines also lack any mechanism for resolving grievances of patients or practitioners.

We believe it is time for the Ministry of Health and Family Welfare (MoHFW) to make use of the experience gained so far to revise the guidelines to address the weaknesses we note above and to establish an ongoing system of evaluation to permit future improvements in the guidelines to make them increasingly comprehensive.

## Importance of infrastructure

The success of telemedicine rests largely on infrastructure. While India has made great progress in the field of telecommunication in the last two decades, a large proportion of rural areas have yet to receive benefits of the digital revolution. According to the Telecom Regulatory Authority of India (TRAI), the government body that regulates all forms of telecommunication, internet subscribers reached 687.62 million as of September 2019 and internet subscriptions reached 52.08 per 100 population [10].

News outlets reported that India had 500 million smartphones in use as of December 2019 [11]. Internet penetration (population that has access to internet), however, amounted to a modest 36% of the overall population, but with only 28% of females in rural India using internet. India’s huge economic, social, gender, and geographical disparities make use of telemedicine across the country challenging. With a sudden rise in internet use due to the pandemic, a spike in data consumption at homes increased at least 15%. This surge likely strains limited resources, including the inadequate optical fibre facilities, a basic necessity in providing internet facilities. Internet speed is slow with the country ranking 131st in mobile internet speed and 65th in fixed mobile broadband speed [12]. Upgrading infrastructure will be essential for telemedicine to succeed [13], especially in rural areas of the country. A massive scale up of capacity and improving infrastructure, preferably by the government, can enable an attractive market for private telecom operators, who will, in turn, facilitate greater and higher quality access to telemedicine services.

## Training doctors in telemedicine

In 2016, the American Medical Association encouraged its undergraduate and postgraduate accrediting bodies to include core competencies for telemedicine in their programs. Medical education regulators in India should learn a lesson from the pandemic situation and also include such competencies in their programs. The American Telemedicine Association, as well as other medical professional bodies, have sub-specialty-specific guidelines for telemedicine services; India should act similarly to broaden the scope of telemedicine in the country [14]. Medical practitioners’ responses to short courses and online certificates and diplomas in telemedicine already indicate these to be increasing in popularity [15].

The effectiveness of telemedicine depends on the practitioners’ competence in specific skills, some of which are different from those required for a traditional face-to-face medical system. Thus, we urge adding new forms of preparation for doctors-in-training as well as for those already in practice. Most Indian doctors lack competencies specific to telemedicine. Among competencies that will be needed for effective digital communication and good 'web-side' manners include those for effective remote examination, group interactions, handling of emergent situations, empathetic communication, interpersonal skills, and troubleshooting. Incorporating telemedicine in undergraduate and postgraduate medical education will be essential for India to be able to scale up telemedicine services [14]. The Telemedicine Society of India has been working assiduously to emphasize the importance of telemedicine, to update information periodically, and to sponsor training conferences for medical and non-medical professionals. It also publishes a regular newsletter highlighting advances in and applications of telemedicine in India [16].

## The Indian telemedicine experience during COVID-19: public and private

On 9 August, 2020 the government of India introduced its telemedicine service, *eSanjeevani*, as part of its ‘Digital India’ initiative. During the COVID-19 pandemic, practitioners have used video conferencing to diagnose and treat patients in geographically diverse locations [17]. The platform currently permits two types of telemedicine services: Doctor to Doctor (*eSanjeevani*) and Patient-to-Doctor (*eSanjeevani* OPD) [18]. These services are part of the larger government scheme to connect larger hospitals to smaller health centres in remote areas. As it expands, medical college hospitals and large government hospitals in the States would act as ‘hubs’ to provide tele-consultation services to ‘spokes’, or primary health care centres (a “hub and spoke” model) [17]. India has relied on this model for providing non-COVID essential healthcare, with approximately 3 million consultations on the *eSanjeevani* platform as on 17 March 2021 [18].

We documented the telemedicine experience of a tertiary diabetes super-speciality hospital in Chennai, in the Southern state of Tamil Nadu, as experienced by one of the authors (VM)**.** Diabetes, a chronic condition, requires patients to frequently visit the hospital for treatment. During the lockdown, with the implementation of telemedicine guidelines, the hospital started offering these services at more than 50 diabetes centres in 32 cities across India. Consultant physicians accessed an already available cloud-based electronic medical record system on their smartphones or computers. They conducted a total of 2864 teleconsultations between April and December 2020, all after seeking consent of the patient and enquiring about each person’s condition and symptoms. After the consultation, physicians sent prescriptions to their patients by email. Many patients expressed satisfaction with the shift to telemedicine as it saved their time, but a few reported that they missed the personal interaction of a face-to-face consultation [19].

## Way forward: exploring opportunities

The positive externalities of investing in and broadening the scope of telemedicine in a resource-poor country like India are many. While the COVID-19 pandemic is ongoing, many cases of non-communicable and other diseases that cause more than 60% of natural deaths in Indians can be addressed using a robust telemedicine infrastructure [20].

The skewed healthcare force distribution in India, where 60% of the force caters to 30% of the population that lives in urban India; with an urban to rural doctors ratio at 3.8:1, can be somewhat mitigated by effective and accessible telemedicine programs [21]. More than 50% of healthcare practitioners in rural India do not have any formal training and with poor access to formal healthcare facilities in vast swathes of rural India, quackery remains a major issue in healthcare [22]. Remote access to Registered Medical Practitioners, or those who have a MBBS degree, using telemedicine can reduce widespread quackery. Telemedicine can bring quality health care, including specialists, to a large population through smartphones. Success will, however, be limited unless India also effectively addresses the country’s social determinants of health like gender inequality, education status, gender disparity in education, women’s participation in labor force and others [23].

In this era, it should be possible to integrate telemedicine with Artificial Intelligence technology, to enhance the quality of healthcare and create new models of care [24]. India is already experimenting with these elements in many initiatives. In Madhya Pradesh, a large state in India, the state government has linked its *eSanjeevani* telemedicine platform to about fifty Primary Health Centres (PHCs) for patient follow-up and care; these centres depend on tertiary care hospitals several hundred kilometres away. With guidance of local medical officers, patients have been seeking consultation from experts. By August 20, 2020, five days since the rollout on August 15, about 1000 patients availed themselves of service. The State plans to extend the program to all its 11,000 PHCs [25].

Data privacy and security protections need to be strengthened in India. The Parliament tabled a Personal Data Protection Bill in December 2019 that would set up a Data Protection Authority and enable imposition of heavy penalties for violations of the law about storing of data and require consent from individuals before accessing their data [26]. A German security firm, Greenbone Networks, have reported several health data breaches and leaks in India. It was a February 2020 report of a leak of patient data from India, including X-ray images, names, ailments, treating physicians, which pointed to gaps in the confidentiality and data protection system [27]. Use of third-party apps (applications on computers, tablets, and telephones) to transmit personal information would make users of telemedicine wary. Strengthening of protections will be essential to success.

This pandemic has boosted initiatives to expand telemedicine. The COVID-19 situation has opened a so called ‘Overton window’ of political possibility, a wider range of options the public is willing to consider and accept. This opening should trigger policy formulation followed by legislation to codify new policies.

## Conclusion

The ongoing efforts of government for expanding use of telemedicine in the face of the COVID-19 pandemic, including the *eSanjeevani* experience, along with private sector initiatives, offer promise for mitigating the dire limitations of healthcare in India. These telemedicine programs should be drawn into the mainstream even after the pandemic crisis, to improve access, equity, training, and quality of Indian health services. Success will require prioritization of short, medium, and long term goals. In the longer term, improving internet infrastructure is essential. Government programs to extend optic fibre to remote areas need to be coupled with linking the smallest administrative and healthcare units like the Public Health Centres (PHCs) and Health & Wellness Centres (HWCs), with larger hospitals and medical college hospitals. For the medium term, objective evaluation is essential. It is timely to evaluate functioning telemedicine programs, both public and private, including the examples of *eSanjeevani* mentioned above. Evaluation results should inform evolution of the guidelines and of the legal framework. In the short term, it is training of medical practitioners that merits urgent attention. Telemedicine ‘crash courses’ or continuing medical education modules can raise awareness among them and help practitioners to keep abreast with technological, ethical, and legal concerns and advances. Gains in use of telemedicine in India are likely to persevere and provide an enabling environment for a more robust healthcare delivery system.
